# Ethical issues in communication in a tertiary oncology center: exploratory survey

**DOI:** 10.3389/fpsyg.2025.1576369

**Published:** 2025-06-06

**Authors:** Elena Ruggiero, Stefania Schiavon, Silvia Stragliotto, Ivan Gallio, Fabio Formaglio, Marina Lorusso, Alessandra Feltrin, Marco Maruzzo, Antonella Brunello

**Affiliations:** ^1^Pain Therapy and Palliative Care Unit, Veneto Institute of Oncology IOV–IRCCS, Padua, Italy; ^2^Department of Oncology, Oncology Unit 3, Veneto Institute of Oncology IOV-IRCCS, Treviso, Italy; ^3^Hospital Psychology Unit, Veneto Institute of Oncology IOV–IRCCS, Padua, Italy; ^4^Department of Oncology, Oncology Unit 1, Veneto Institute of Oncology IOV-IRCCS, Padua, Italy

**Keywords:** communication, bad news, oncology, ethics, ethical issue

## Abstract

**Background:**

Despite evidence of its importance, communication in oncology remains a critical challenge, especially in case of bad news. The doctor-patient relationship is often strained by time limitations, emotional challenges, and cultural or ethical dilemmas surrounding end-of-life discussions. This study examines barriers to effective communication at the Veneto Institute of Oncology (IOV), focusing on time constraints and emotional difficulties in clinical practice. It aims to identify factors hindering timely and effective discussions on bad news and end-of-life issues, the primary participants in such conversations, and reasons for delays in addressing sensitive topics.

**Materials and methods:**

An anonymous questionnaire was completed by 43 attending physicians from Oncology and Haemato-oncology departments at the IOV, with 69.8% of the respondents being women. The majority of the respondents were under 40 years of age. Data on demographics, roles, and communication practices were analysed to identify behavioral patterns.

**Results:**

Most respondents (65.1%) prioritized communicating bad news to patients rather than caregivers. Time constraints were the most reported barrier (40%), followed by emotional distress, fear of demotivating patients, and insufficient training. Despite challenges, 76.7% felt confident in shared decision-making with patients.

**Conclusion:**

The study highlights the need for structured communication training and better resources to address time and emotional barriers, to enhance patient autonomy and to reinforce doctor-patient relationships in end-of-life care.

## 1 Introduction

### 1.1 Sustainability of contemporary socio-health systems

The exponential increase in patients and services provided by the National Health Service (NHS) leads to reduced time for each consultation, risking the quality of the doctor-patient relationship. Furthermore, the tendency to view mortality as an “enemy” to defeat may lead to disproportionate interventions and suggest that happiness and health must coincide. Today, medicine is expected to address not only physical and emotional well-being. This cultural expectation contributes to approach social issues and the enhancement of healthy individuals’ characteristics (Callahan, 2009; Viafora, 2023).

The sustainability of contemporary socio-health systems represents a complex and urgent issue, intertwining various factors: the role of health services in respecting human dignity, differing evaluation criteria for health choices, and emerging cultural challenges surrounding the very notion of health. Ethically, justifying health choices must consider both medical and economic-organizational criteria (Callahan, 2009). In a context where resource scarcity has become a structural condition, the need arises to shift from viewing limitations as failures to recognizing them as opportunities for prioritization (Viafora, 2023).

### 1.2 Communication in oncology

Managing doctor-patient communication in oncology is one of the most delicate challenges healthcare professionals faces (Virginia, 2006; Berardi et al., 2024). The delivery of bad news is not an isolated moment but a continuous process requiring constant support and open dialogue throughout the care journey. As a result, the relationship is often deliberative, based on the premise that a patient’s values should be discussed and challenged if necessary. This relationship demands that doctors be available to address patients’ questions and concerns, offering emotional support at every treatment stage (Viafora, 2023; Virginia, 2006; Postavaru et al., 2023; Mori et al., 2022).

In this context, “time” is crucial. As chronic and incurable conditions take up more space due to increasingly specialized therapies, it is difficult to reconcile the need to see more patients with the time required to foster an effective doctor-patient relationship, especially during difficult communications. Reducing time for communication might disadvantage the doctor, the patient, and the NHS. It can be un-ethical for doctors discuss end-of-life issues in a timely manner. Such circumstances may influence an oncologist’s approach to disease progression or treatment cessation (Tronto, 1993; Tusino et al., 2019; Tymieniecka and Agazzi, 2001; Ricoeur, 2006).

The dynamics of the doctor-patient relationship are aptly represented in the model of “care ethics,” which emphasizes a symmetrical relationship where one party commits to helping the other meet needs that they cannot fulfill alone. For instance, moving beyond paternalistic approaches, actively involving the patient is crucial for reinforcing their autonomy. Illness creates dependency, challenging the patient’s sense of integrity and control. Therefore, actively engaging the patient in treatment decisions—informing them about options, listening to their preferences, and collaborating on informed choices—is essential. Achieving a balance between honesty and sensitivity is vital; providing accurate information while maintaining empathy is crucial (MP Project, 2002; Medicine AFA-AFEF of I, 2002).

In a diverse and multicultural society, communication must account for cultural and linguistic differences, adapting messages to meet individual patients’ needs for better understanding.

The doctor-patient relationship can be viewed as an alliance where both parties commit to upholding shared decisions on care goals and treatment limits (Tusino et al., 2019; Tymieniecka and Agazzi, 2001; Ricoeur, 2006). Delivering bad news entails significant challenges, from managing personal emotions to effective patient communication and complex therapeutic decisions. In brief, adequate training and ongoing support are essential for effectively navigating these challenges (Baile et al., 2000).

Several factors often limit the effectiveness of bad news communication in oncology (Eggly et al., 2006):

- Lack of specific training: Communication requires specialized skills. Doctors must clearly convey complex information, ensuring understanding without causing confusion or panic.
- Compassion fatigue and burnout: The emotional toll can lead to burnout among doctors, highlighting the need for emotional and professional support (Santos et al., 2024).
- Managing patient emotional reactions: Addressing intense emotions from patients and families can be challenging. Doctors must be prepared to respond empathetically to various reactions, such as shock, sadness, and denial.
- Time constraints: Many doctors work in busy, high-pressure environments, which can limit the time available for thorough and empathetic communication.
- Limited resources: A shortage of personnel and psychological support services restricts healthcare professionals’ ability to provide adequate emotional and informational support during bad news delivery.
- Cultural and language barriers: Differences in language and cultural norms can hinder the effective transmission and understanding of crucial information.

Additionally, providing patients with adequate emotional, psychological, and practical support is essential to navigate the challenges of illness (Tymieniecka and Agazzi, 2001; Tronto and Fisher, 1990). Ineffective communication can heighten feelings of anxiety, loss of control, and isolation, complicating decision-making processes.

### 1.3 Bioethical issues

The ethical challenges in communication within oncology present complex dilemmas that require a delicate and careful approach from healthcare professionals involved. Respecting fundamental ethical principles, such as patient autonomy, beneficence, and non-maleficence, is essential to ensure empathetic, respectful, and ethically responsible communication throughout the cancer patient’s care journey (Tuca et al., 2021; Ullrich et al., 2020; Costello, 2000).

Healthcare professionals have a duty to provide accurate and complete information to patients, respecting the principle of autonomy and the right to be fully informed about their health condition. However, there is also the risk of causing emotional and psychological harm by conveying information that may be too traumatic or difficult to handle. In case of negative prognosis healthcare professionals must convey the truth with empathy and tact, trying to alleviate the emotional pain of the patient and their family.

In this context, the appropriateness of communication methods is crucial (Stone et al., 2023): healthcare professionals should adopt communicative approaches that are sensitive to the emotional needs of the patient, offering adequate psychological support and connecting the patient to individualized support resources. From a regulatory standpoint, Law 219/2017 outlines the contours of the care relationship: our institution regularly organizes courses and meetings.

### 1.4 Information overload and conspiracy of silence

Information overload happens when a person is exposed to more information than they can process effectively. This can negatively impact mental health, causing feelings of overwhelm, anxiety, or stress. In oncology communication, it arises when patients and families receive excessive details about their diagnosis, treatment, or prognosis without adequate time or resources to process it or cope emotionally (Eraslan and İlhan, 2023; van Ravensteijn et al., 2023; Lillie et al., 2023).

To address this, communication should balance clarity and relevance, avoiding unnecessary information while providing emotional support. Current legislation (Law 219/2017) also ensures patients’ right not to know, requiring this choice to be recorded and shared with the care team (Viafora, 2023; Tusino et al., 2019; Jensen et al., 2014). However, how this is recorded and communicated to teams remains underexplored.

Ethical Arguments in Favor of Complete Patient Information

- Respect for Autonomy: the fundamental ethical principle is patient autonomy, which emphasizes the patient’s right to be fully informed and to be involved in decisions. Providing complete and accurate information about a cancer diagnosis respects the patient’s self-determination and informed decision-making (Viafora, 2023; Tusino et al., 2019).
- Beneficence: complete information allows patients to understand their condition, to participate actively, and to adopt behaviors and decisions that can improve their prognosis (Viafora, 2023; Tusino et al., 2019).
- Justice: to ensure that all patients have access to the same opportunities for care and that no patient is treated discriminatorily or unfairly (Viafora, 2023; Tusino et al., 2019).
- Prevention of Harm: Omitting crucial information can negatively affect the patient’s treatment decisions, compromising treatment effectiveness and worsening outcomes. Furthermore, a lack of transparency regarding their health can cause anxiety, stress, and emotional distress (Viafora, 2023; Tusino et al., 2019).
- Trusting Relationship: Open and transparent communication between doctor and patient is essential for maintaining a strong and positive relationship (van Ravensteijn et al., 2023).

Ethical Arguments for Partial Patient Information (PPI), underlining that this choice must always be agreed upon with the patient, as required by Law 219/2017:

- Minimization of Emotional Harm: providing all information at once could cause significant emotional and psychological trauma. In this scenario, according to certain perspectives, particularly those with a paternalistic approach. Providing PPI can allow the patient to gradually process the news, reducing the risk of severe immediate emotional reactions (Viafora, 2023; Tusino et al., 2019).
- Protection of Optimism and Hope: PPI can help preserve the patient’s optimism and hope, allowing them to maintain a positive outlook on their health and healing possibilities. In some cases, knowing all the details about a cancer diagnosis might lead to despair and a loss of hope, compromising their resilience and willingness to fight the disease (Viafora, 2023; Tusino et al., 2019).
- Preservation of the Doctor-Patient Relationship: In some cases, providing PPI may be misinterpreted to protect the doctor-patient relationship. If the doctor believes that communicating certain information could irreparably damage the trusting relationship or the patient’s confidence in treatment, they may choose to delay or limit the disclosure of such information (Viafora, 2023; Tusino et al., 2019).
- Minimization of the Risk of Hasty Decisions: Providing PPI can give patients time to process the news and consult with family members or advisors before making important treatment decisions, thereby reducing the risk of hasty or irrational decisions made during periods of significant emotional stress (Eraslan and İlhan, 2023).

The purpose of the survey was to provide a snapshot of doctor-patient communication during the journey of metastatic cancer patients within our center and to analyze, from an ethical perspective, the main vulnerabilities that must be addressed through enhancement programs and specific training. This aspires to ensure not only the best technical and scientific care but also comprehensive patient management, integrating the oncological pathway with early palliative care.

## 2 Materials and methods

The study in question originates within the Advanced Course in Bioethics at the University of Padua (academic year 2023/2024). The objective of the survey was to assess, through the completion of an anonymous questionnaire given to the medical staff of the Medical Oncology and Haemato-oncology departments of the Veneto Institute of Oncology (IOV), the actual difficulties encountered in clinical practice regarding the communication of bad news in relation to the time allocated for visits, the demands of patients and caregivers, and the risk of overwhelming them with information. Additionally, the aim is to investigate who the main interlocutor was (patient/caregiver) and the reasons that often lead to addressing end-of-life issues later than necessary.

We conducted a survey targeting the 65 staff physicians of the institute of the Medical Oncology and Haemato-oncology departments of the Veneto Institute of Oncology (IOV). The study involved the distribution of a brief anonymous questionnaire, available both online via Google Forms and in paper format. The inclusion criteria were that participants be currently practicing physicians in one of the aforementioned departments and who voluntarily agreed to complete the questionnaire. Responses were collected anonymously to encourage honest and open feedback.

### 2.1 Questionnaire structure

As shown in Table 1, the questionnaire consisted of two main sections:

- Demographic Data: This section collected basic information about the participants, including gender, age group, professional role, years of service, specialization, and primary work setting (outpatient clinic, day hospital, or inpatient ward).
- Communication and Emotional Aspects: This section addressed the participants’ experiences and perceptions regarding the communication of bad news to patients and caregivers, the challenges they face, and the emotional reactions they experience. Questions also explored the frequency of discussing specific topics (such as end-of-life care and palliative care) with metastatic oncology patients and assessed the main barriers to effective communication.

**Table 1:** Questionnaire proposed to oncologists and haematologists of the Veneto Oncology Institute.

Personal information
Gender
Age group
Professional role
Years of service
Achieved specialty
Predominant work setting: outpatient clinic/day hospital/ward
Communicative aspects
With whom do you think it is more appropriate to share negative prognostic information first: patient/caregiver?
How often (never, sometimes, often, always) do you address these topics with metastatic cancer patients at the time of their initial care, at the time of disease progression, and at the time of definitive treatment cessation transitioning to exclusive palliative care: Advance care planning End of life and sedation Impact of treatments on quality of life Activation of concurrent/exclusive palliative care Medically assisted death
In clinical practice, what factor do you consider to be the most limiting in communication with the patient at the time of initial care for the metastatic cancer patient, at the time of disease progression, and at the time of definitive treatment cessation transitioning to exclusive palliative care: Lack of time Fear of demotivating or frightening the patient At the request of the caregiver I do not feel capable of doing it I want to avoid information overload
How much is the lack of time a source of discomfort for you on a scale from 0 to 10 in communication with the patient (if you indicated “lack of time” in the previous questions)?
Emotional reactions
What are the emotional reactions that most often arise from these communications in your personal experience? Sense of professional inadequacy or defeat Excessive empathy and identification with the patient, accompanied by anxiety or fear Awareness of having made the best choice for the patient within a shared care planning context I need to address the situation with the support of a colleague

### 2.2 Data collection and analysis

The responses were collected over a month and then subjected to statistical analysis. Frequencies and percentages were calculated to describe the distribution of responses. We also conducted comparative analyses based on the participants’ demographic and professional training variables. The SPSS 24 software package for Windows (SPSS, Inc., Chicago, IL, United States) was used to manage the database and perform the statistical analysis. Nominal qualitative variables were analyzed using the Phi coefficient, Cramér’s V, and Chi-square test or Fisher’s exact test, depending on sample size. The significance level was set at *p* < 0.05 for all tests.

### 2.3 Ethical considerations

Participation was voluntary, and all data were collected anonymously to ensure the confidentiality of the participants. The study was conducted in accordance with ethical guidelines for research involving healthcare professionals.

## 3 Results

A total of 43 individuals out of the 65 subjects to whom the study was proposed participated in the survey, of which 30.2% were male. The majority of the sample was aged between 31 and 40 years (23 subjects, 53.5%). Of the respondents, 74.4% were oncologists (32 subjects), while 14% were haemato-oncologists (6 subjects).

Most of the respondents had 0–5 years of service (37.5%) or 6–10 years of service (34.9%), while 28% had more than 11 years of service. The predominant work settings were the outpatient clinic (60.55%) and the inpatient ward (27.9%).

Regarding the question about with whom it is most appropriate to share bad news first, 34.9% indicated the caregiver, while 65.1% indicated the patient. There were no statistically significant correlations between the choice of first interlocutor (patients or caregiver) and demographic or professional variables (Chi-square test for decades of age *p* = 0.866, physicians vs. residents *p* = 0.398, work seniority *p* = 0.796, oncologists vs. hematologists *p* = 0.069, inpatients vs. outpatients vs. day-hospital *p* = 0.393).

**Figure 1:**
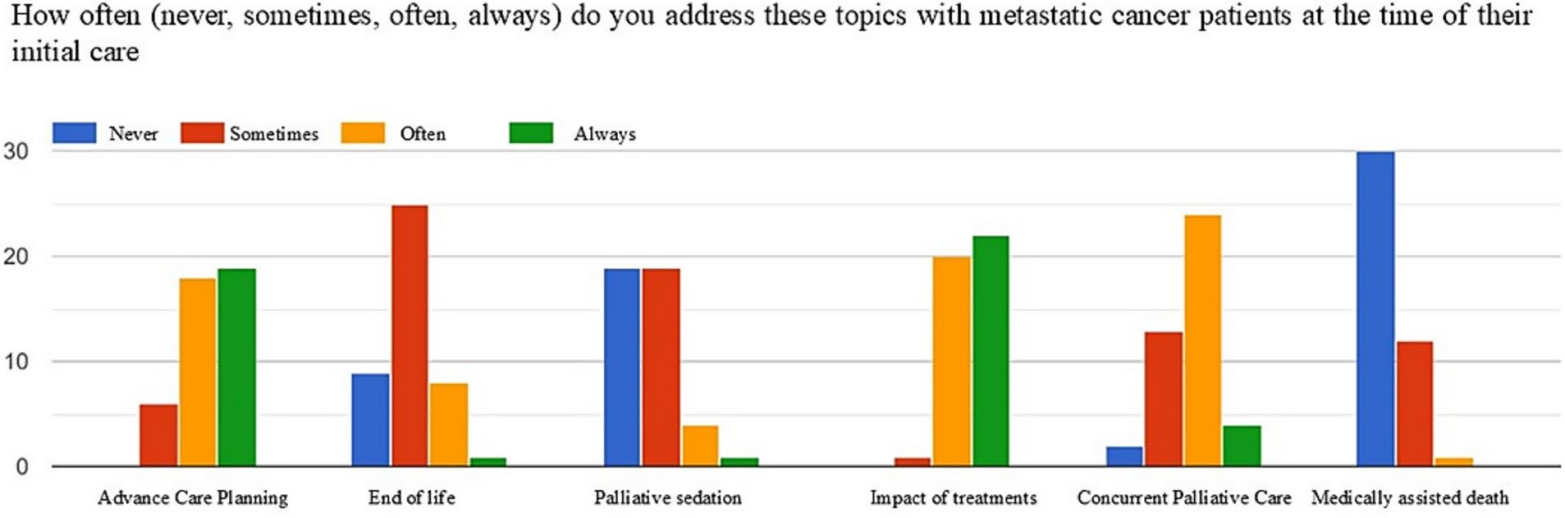
Frequency of addressing the topics of ACP, end-of-life, sedation, the impact of care on quality of life, activation of simultaneous palliative care, and medically assisted death at the time of taking charge of metastatic oncology patients.

**Figure 2:**
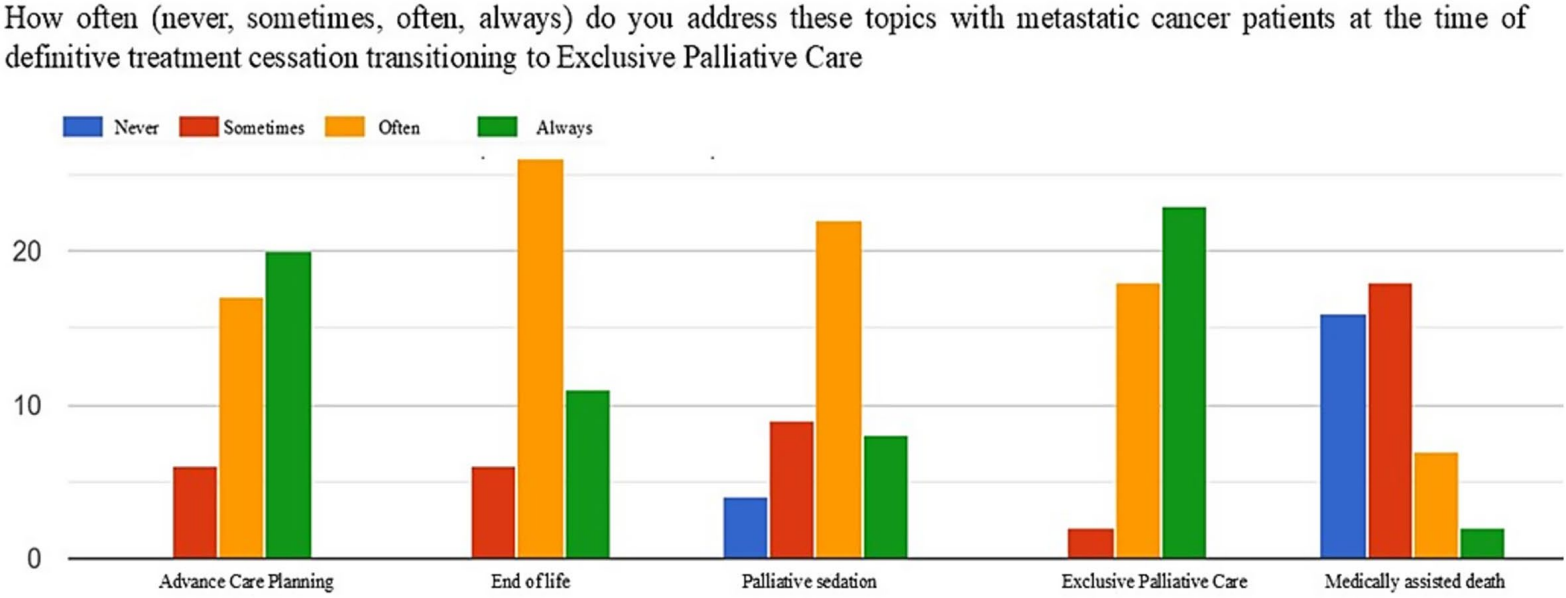
Frequency of addressing the topics of ACP, end-of-life, sedation, the impact of care on quality of life, activation of simultaneous palliative care, and medically assisted death at the time of disease progression.

**Figure 3:**
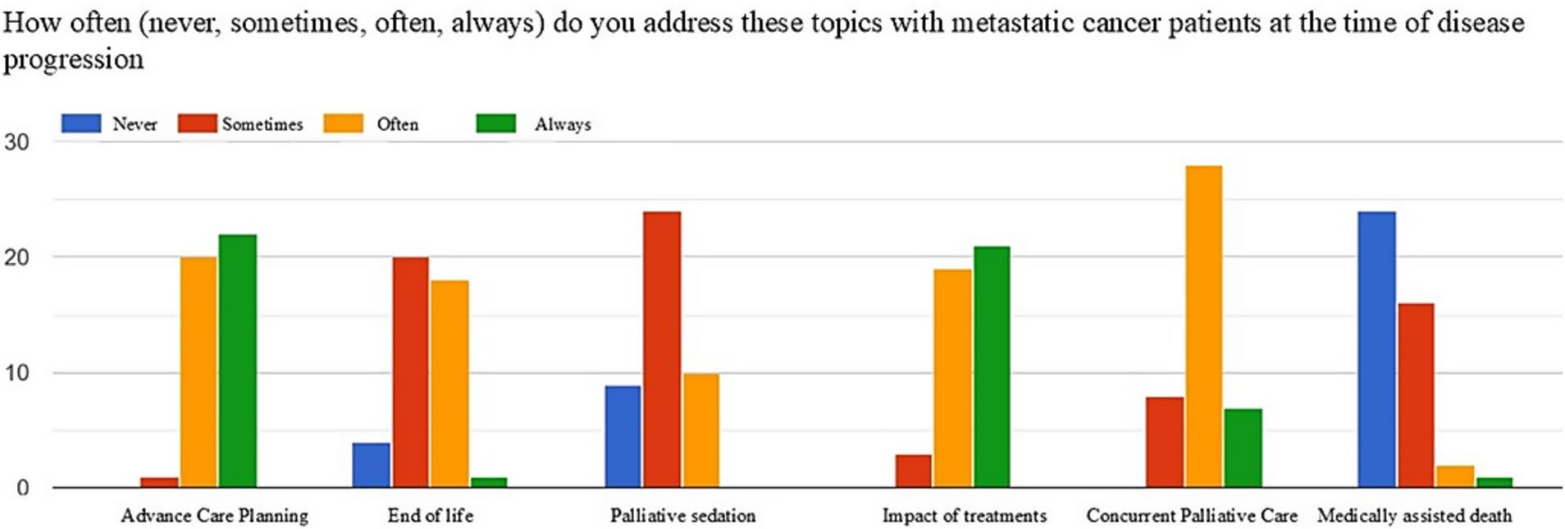
Frequency of addressing the topics of ACP, end-of-life, sedation, activation of exclusive palliative care, and medically assisted death at the time of the definitive suspension of active oncological treatments.

Figures 1–3 show the frequency (never, sometimes, often, always) with which the topics of advance care planning (ACP), end-of-life and sedation, impact of treatments on quality of life, activation of simultaneous/exclusive palliative care, and medically assisted death are addressed at key moments in the oncology care pathway, namely patient intake, disease progression, and the final discontinuation of treatments with referral to exclusive palliative care.

At the time of patient intake, the majority of respondents often (42%) or always (44%) discussed the concept of ACP with the patient, as well as the impact of treatments on quality of life (often 46%, always 51%). Conversely, end-of-life topics, sedation, and medically assisted death were addressed less frequently by most respondents (end of life: never 51%, sometimes 20%; sedation: never 44%, sometimes 44%; medically assisted death: never 70%, sometimes 27%).

At the time of disease progression, most respondents revisited the ACP (often 46%, always 51%), focusing attention on the impact of treatments on quality of life (often 44%, always 48%). End-of-life and sedation topics were addressed more frequently, while most respondents did not discuss medically assisted death with the patient (end of life: often 46%, sometimes 41%; sedation: often 10%, sometimes 55%; medically assisted death: never 55%).

At the time of the final discontinuation of active treatments and transition to exclusive palliative care, the majority of respondents more frequently addressed end-of-life topics (often 60%, always 11%) and sedation (often 51%, always 18%). Similarly, 95% of the sample shared with the patient the goals of exclusive palliative care (often 41%, always 53%).

**Figure 4:**
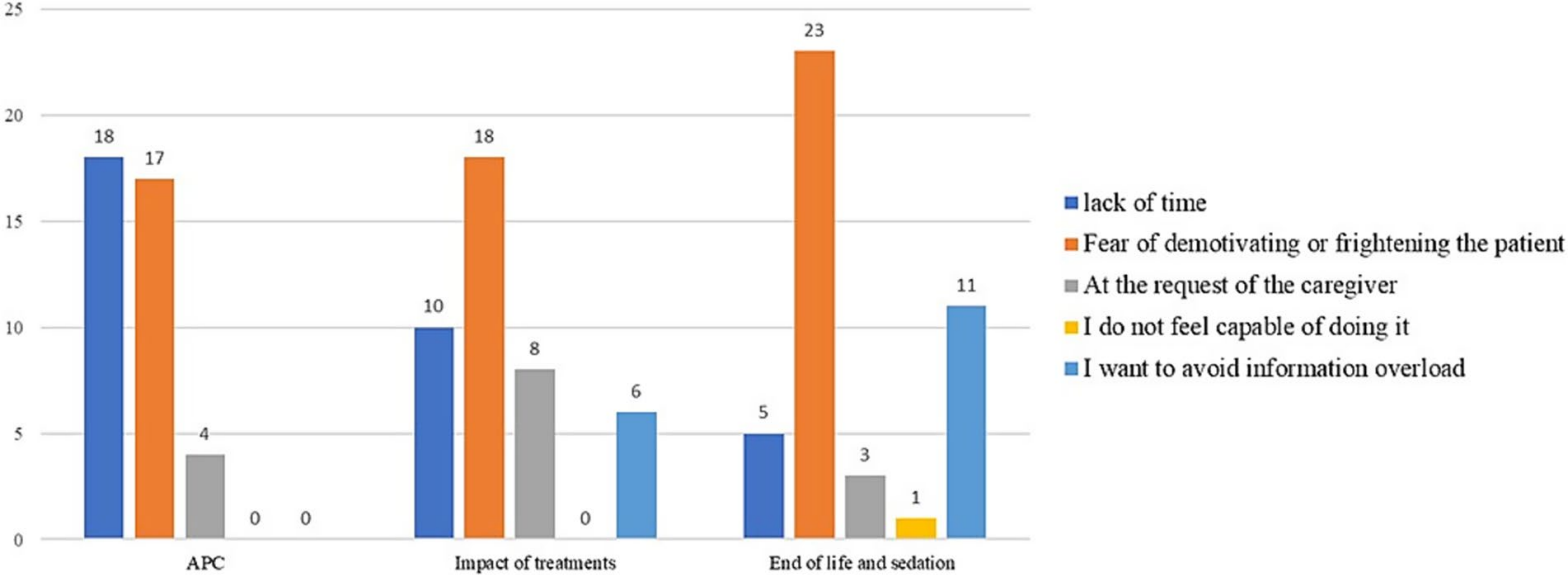
Limiting factors in communication regarding ACP, the impact of care on quality of life, end-of-life, and sedation at the time of taking charge of metastatic oncology patients.

As shown in Figure 4, at the time of intake for a metastatic oncology patient, time is the most limiting factor in communications regarding ACP (41%). However, when it comes to communications about the impact of treatments and end-of-life issues, the fear of demotivating the patient is equally or more significant (32% for treatment impact and 48% for end-of-life issues, respectively).

**Figure 5:**
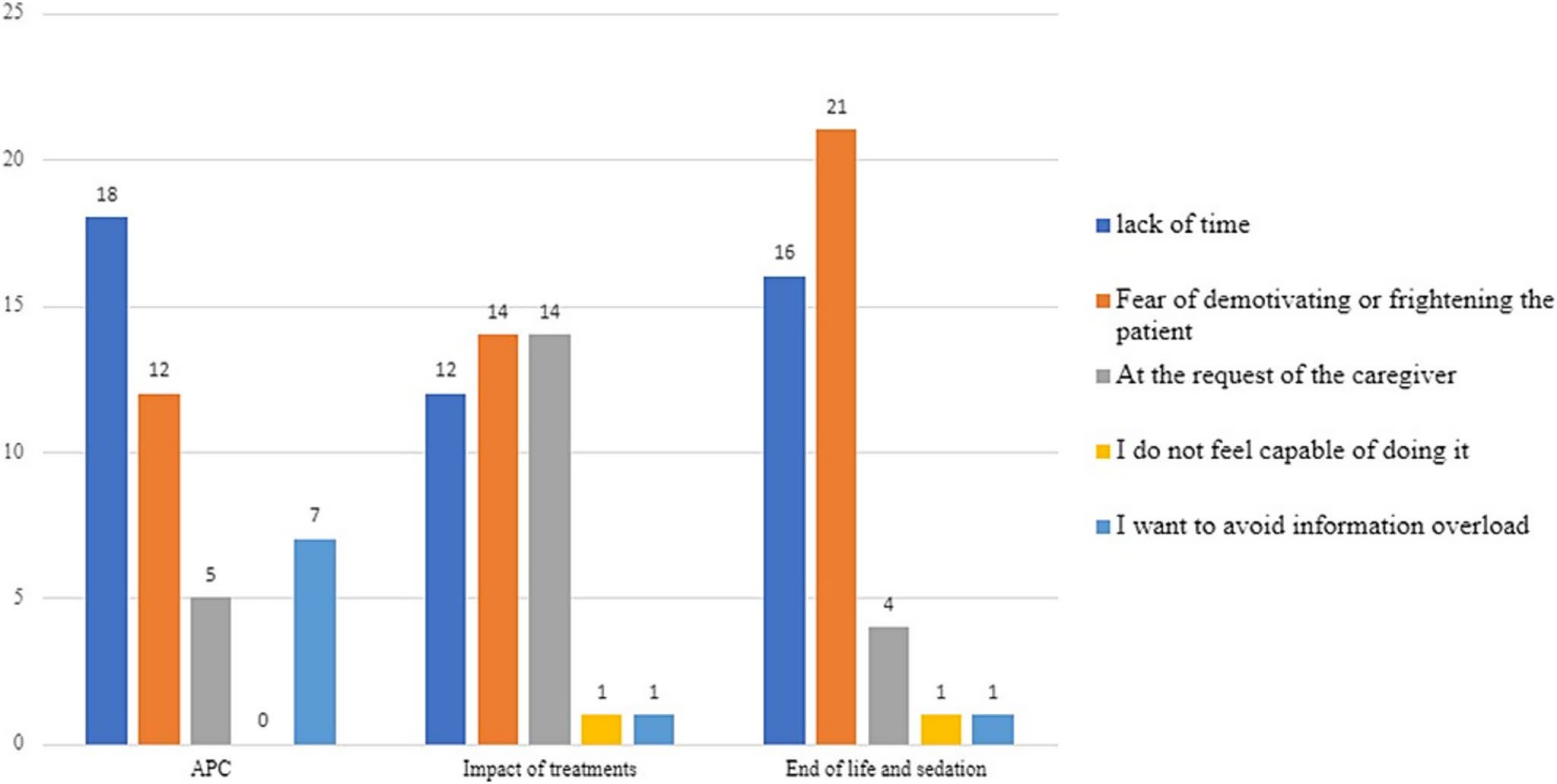
Limiting factors in communication regarding ACP, the impact of care on quality of life, end-of-life, and sedation at the time of disease progression.

At the time of disease progression (Figure 5), time remains the most limiting factor in shared care planning (42%). However, regarding communications about the impact of treatments and end-of-life topics, the fear of demotivating the patient is perceived as the greatest obstacle (42% for treatment impact and 53% for end-of-life issues, respectively).

**Figure 6:**
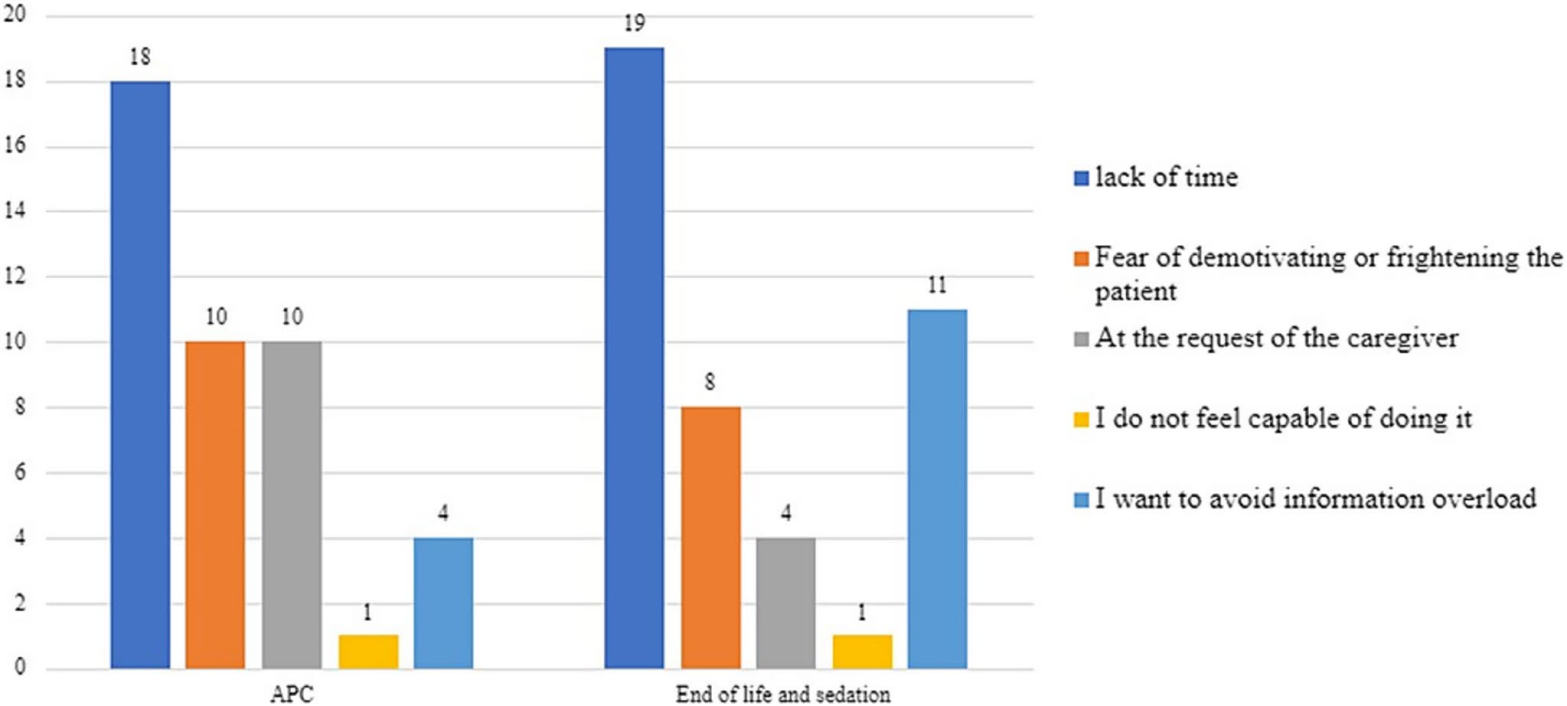
Limiting factors in communication regarding ACP, end-of-life, and sedation at the time of the definitive suspension of active oncological treatments.

At the time of the final discontinuation of active oncological treatments (Figure 6), time is perceived by about half of the respondents as the most limiting factor: 42% for ACP and 44% for end-of-life topics and palliative sedation.

A clear significant correlation emerges between the predominant work setting and the frequency with which ACP is redefined, with a greater emphasis placed on it in outpatient clinics, where continuity of care with the treatment team is higher compared to the inpatient ward (p 0.019, Pearson Chi-Square 11.778).

Oncologists are more inclined to discuss end-of-life matters with patients at the time of treatment discontinuation compared to haemato-oncologists (p 0.29, Pearson Chi-Square 10.768).

In general, the availability of very limited time for managing visits and conversations is a significant source of distress (> 6/10) for more than 80% of respondents, and even higher, above 8/10, in 62.8% of cases: efforts from policy makers and stakeholders are needed to balance the time between the clinical cure and the conversation.

From the analysis of emotional reactions resulting from the communication of bad news, the vast majority of respondents are aware that they made the best decision for the patient within the context of shared care planning (76.7%), while only a minority experience a sense of professional inadequacy or defeat (9.3%) or an excess of empathy and identification with the patient, leading to anxiety or fear (11.6%).

## 4 Discussion

### 4.1 The ideal of communication time as care time

The first observation that emerges from our study is the low participation in the survey: only about 66% of those interviewed participated. Although the literature already highlights a limited awareness of the role and tools of bioethics (Tuca et al., 2021), non-participation may reflect limited engagement with ethical discussions in clinical practice; this result prompted us to consider the possibility of specific training in this area, as well as in proactive communication. Such training aims to enhance this sensitivity, which should become a foundational aspect of clinical practice and be safeguarded alongside technical and scientific training. In particular, the low participation in the study mainly concerns oncologists dealing with conditions for which new therapeutic strategies have significantly prolonged survival expectations, such as breast cancers, thereby delaying the time-point for delivering bad news. The question we ask ourselves in light of this data is how this attitude should be modulated over time and how to enable the oncologist to identify the right moment to introduce topics related to end-of-life care for long survivors.

Indeed, limited time for patient visits can negatively impact communication between healthcare professionals and patients, making it difficult to address concerns, provide complete information, and offer adequate emotional support. The data from our survey confirms that the limited time available for managing visits and conversations is a source of significant distress for the majority of respondents, compromising essential communications, such as those regarding ACP. The ACP is a critical element in building a therapeutic alliance and shared decision-making, where patient involvement is one of the most important tools physicians have to strengthen the patient’s sense of autonomy in the face of uncertainty and loss of control due to illness (Berardi et al., 2024; Tymieniecka and Agazzi, 2001; Ricoeur, 2006; Tronto, 2014).

Several aspects of communication are negatively impacted by limited time, as highlighted in the literature (Eggly et al., 2006; SICP, n.d.):

- Superficiality: The lack of time can result in a tendency to focus solely on the most urgent aspects of the patient’s condition, neglecting emotional concerns and psychological needs.
- Reduced ability to delve deeper: Limited time may prevent healthcare professionals from exploring patient concerns in detail, leaving communication superficial, and making patients feel unheard or misunderstood.
- Incomplete information: Time constraints can lead to fragmented communication, resulting in the patient’s incomplete understanding of their condition.
- Risk of misunderstandings.
- Less room for emotional support: Limited time reduces the ability to provide emotional support, causing communication to center primarily on medical aspects, overlooking the emotional and psychological needs of both patients and their families.

The lack of time compromises the quality of the doctor-patient relationship. It is essential to reflect on how to balance the increasing demands on the NHS, rising life expectancy due to new therapies, and the scarcity of resources and time in the healthcare sector. Balancing these aspects while respecting patient dignity and physician professionalism requires ongoing commitment from national entities and the government. To conclude, health is increasingly recognized as a central component of well-being in our socio-cultural context. Targeted training in structured communication methods—such as the SPIKES protocol and shared decision-making frameworks—constitutes a key strategy for enhancing communication in oncology. Educational programs incorporating simulation-based learning, role-play, and blended learning approaches have been shown to improve healthcare professionals’ abilities to address patients’ emotional needs, deliver complex information transparently, and build therapeutic trust.

### 4.2 Ethics and the “conspiracy of silence”

Our data supports the idea that informing the caregiver before the patient is still common practice (35% of the sample). This is often done with the aim of protecting the patient but contradicts best medical practices, even from a legal standpoint. Such protective attitudes, akin to paternalistic ethics where the patient is not fully regarded as a decision-maker, can lead to a phenomenon called the “conspiracy of silence.” This controversial practice involves hiding crucial information about diagnosis, prognosis, or treatment from the patient, often with the involvement of family and the medical team. Unless explicitly agreed upon with the patient, this practice undermines the patient’s autonomy, favoring a questionable use of the principle of non-maleficence (Viafora, 2023; Tuca et al., 2021; Ullrich et al., 2020; Costello, 2000).

Common justifications for this partly omissive attitude include:

- Preserving optimism and hope: Maintaining a positive outlook during treatment.
- Minimizing emotional harm: Aiming to protect the patient’s quality of life by reducing emotional distress.
- Protecting the doctor-patient relationship: Some believe that revealing negative information could irreparably harm the trust between the patient and the physician.

Conversely, arguments against the conspiracy of silence include:

- Violation of patient autonomy: Concealing vital information undermines the patient’s right to make informed decisions about their care.
- Lack of transparency: Hiding information erodes trust in the doctor-patient relationship and compromises patient confidence in the care they receive.
- Potential long-term harm: Failing to provide critical information can harm the patient’s long-term physical and psychological well-being by preventing them from making informed choices.
- Disrespect for patient dignity: Concealing information shows a lack of respect for the patient’s dignity and autonomy, treating them as incapable of handling the truth about their condition.

The widespread preference for discussing bad news with the caregiver first, which does not vary significantly by age, years of experience, or specialty, suggests that the current outpatient structure may not provide enough time and space for open discussions about ACP with the patient. Ethically, it would be more appropriate to allow the patient to decide what information they wish to receive and how it should be shared with others, in alignment with Law 219/2017. The intention to protect the patient by first informing the caregiver can, in cases where the patient is capable of self-determination, deprive the patient of the right to inform loved ones in a manner and time that they see fit. This infringes upon patient privacy and may damage the trust that is meant to be preserved. Across Western jurisdictions, legislative approaches to advance directives differ considerably, reflecting heterogeneous ethical and legal traditions. In France, the Leonetti (2005) and Claeys-Leonetti (2016) laws provide for non-binding directives and permit continuous deep sedation under defined clinical circumstances. Germany’s 2009 legislation confers binding status to advance directives when they clearly address the medical scenario. The Netherlands and Belgium include advance euthanasia requests within their legal frameworks, the latter permitting validity for up to five years. The United Kingdom, through the Mental Capacity Act 2005, acknowledges legally binding Advance Decisions to Refuse Treatment (ADRT) if formal criteria are satisfied. In the United States, the Patient Self-Determination Act (1990) mandates institutional recognition of advance directives, which vary at the state level and may include Living Wills, Healthcare Powers of Attorney, and POLST forms. Despite variations in enforceability and scope, these legal instruments converge in safeguarding decisional capacity and promoting patient self-determination at the end of life.

As also emphasized by the Code of Medical Ethics (Codice di Deontologia Medica, 2014), doctor-patient communication must always be respectful of the patient’s dignity, privacy, and autonomy. The doctor must listen carefully to the patient’s concerns and respond to their questions transparently and with empathy. In particular, in delicate situations such as a serious or terminal diagnosis, the doctor is required to communicate in a sensitive and appropriate manner, avoiding unnecessary anxiety while providing the patient with realistic information that respects their emotional needs. By strengthening communicative competence, such training enables clinicians to conduct difficult conversations with honesty and empathy, thereby supporting informed decision-making and preserving patient autonomy throughout the continuum of oncological care. In doing so, it significantly reduces the risk of the so-called “conspiracy of silence” and promotes open, respectful communication.

## 5 Conclusions and future implications

Several limitations characterize our survey, in particular the small sample size, single-center design and the response rate. However, the observations that emerge from it confirm the main ethical issues related to doctor-patient communication, particularly in oncology. Furthermore, there are not many studies addressing these aspects, so we believe that, despite being limited to our experience, our conclusions may be useful for evaluating training enhancement programs and for promoting greater integration with bioethics consultants and palliative care specialists in the informational process typical of modern oncology. To foster this relationship while respecting the principles of autonomy, beneficence, and non-maleficence, adequate time and space are essential. Sufficient time benefits the patient by making them feel heard and able to ask questions and gradually absorb information. Relationship as care: it helps the physician to share comprehensive information, avoiding the misleading notion that providing information always leads to understanding.

We hope these data will contribute to efforts within the Oncology Institute to address organizational, spatial, and resource limitations that result in a lack of available time. We will use the data obtained to provide specific training in the areas identified as most deficient and to reshape, where possible, the approach to outpatient activities, with greater integration with the Palliative Care team in the perspective of simultaneity. In the future we aim to repeat the questionnaire after specific training, potentially using innovative tools such as blended learning, and to involve scientific society for a nationwide survey. Owing to its structure, the questionnaire lends itself to implementation in a variety of clinical contexts beyond our institution. The structural constraints associated with the limited time scheduled for patient consultations constitute a systemic issue that extends across the national healthcare system and is similarly observed in international settings. As such, the instrument may provide valuable insights into communication dynamics and organizational challenges common to diverse healthcare environments.

Promoting specialized training in communication strategies, especially considering the increasing diversity of the population, would be highly beneficial: training initiatives that integrate simulation-based activities, role-playing exercises, and blended learning modalities may strengthen healthcare professionals’ competencies in responding to patients’ emotional concerns, delivering complex information with clarity and empathy, and building a foundation of trust. Additionally, adequate training on end-of-life care and medically assisted death would allow professionals to openly and proactively discuss these topics within a comprehensive ACP, free from common cultural preconceptions.

## Data Availability

The raw data supporting the conclusions of this article will be made available by the authors, without undue reservation.

## References

[ref1] Baile W. F. Buckman R. Lenzi R. Glober G. Beale E. A. Kudelka A. P. (2000). SPIKES—A six-step protocol for delivering bad news: application to the patient with Cancer. Oncologist 5, 302–311. doi: 10.1634/theoncologist.5-4-302, PMID: 10964998

[ref2] Berardi R. Parisi A. Maruzzo M. Bellani M. Beretta G. D. Boldrini M.. (2024). Communication in oncology between healthcare providers, patients, the scientific community, and the media: recommendations from the Italian Association of Medical Oncology (AIOM). Support Care Cancer 32:613. doi: 10.1007/s00520-024-08786-8, PMID: 39222131 PMC11369048

[ref3] Callahan D. (2009). La Medicina Impossibile. Le Utopie e Gli Errori Della Medicina Moderna: Dalai Editore.

[ref4] Codice di Deontologia Medica (2014). Available online at: https://portale.fnomceo.it/codice-deontologico/

[ref5] Costello J. (2000). Truth telling and the dying patient: a conspiracy of silence? Int. J. Palliat. Nurs. 6, 398–405. doi: 10.12968/ijpn.2000.6.8.9065, PMID: 12411852

[ref6] Eggly S. Penner L. Albrecht T. L. Cline R. J. Foster T. Naughton M.. (2006). Discussing bad news in the outpatient oncology clinic: rethinking current communication guidelines. J. Clin. Oncol. 24, 716–719. doi: 10.1200/JCO.2005.03.0577, PMID: 16446346

[ref7] Eraslan P. İlhan A. (2023). Cancer information overload and death anxiety predict health anxiety. Eur. Rev. Med. Pharmacol. Sci. 27, 291–298. doi: 10.26355/eurrev_202301_30902, PMID: 36647879

[ref8] Jensen J. D. Carcioppolo N. King A. J. Scherr C. L. Jones C. L. Niederdeppe J. (2014). The cancer information overload (CIO) scale: establishing predictive and discriminant validity. Patient Educ. Couns. 94, 90–96. doi: 10.1016/j.pec.2013.09.016, PMID: 24268921

[ref9] Lillie H. Katz R. A. Carcioppolo N. Giorgi E. A. Jensen J. D. (2023). Cancer information overload across time: evidence from two longitudinal studies. Health Commun. 38, 1878–1886. doi: 10.1080/10410236.2022.2038866, PMID: 35172651 PMC9378766

[ref10] Medicine AFA-AFEF of I (2002). Medical professionalism in the new millennium: a physician charter. Ann. Intern. Med. 136, 243–246. doi: 10.7326/0003-4819-136-3-200202050-00012, PMID: 11827500

[ref11] Mori M. Morita T. Bruera E. Hui D. (2022). Prognostication of the last days of life. Cancer Res. Treat. 54, 631–643. doi: 10.4143/crt.2021.1573, PMID: 35381165 PMC9296934

[ref12] MP Project (2002). Medical professionalism in the new millennium: a physicians’ charter. Lancet 359, 520–522. doi: 10.1016/S0140-6736(02)07684-5 11853819

[ref13] Postavaru G. McDermott H. Biswas S. Munir F. (2023). Receiving and breaking bad news: a qualitative study of family carers managing a cancer diagnosis and interactions with healthcare services. J. Adv. Nurs. 79, 2211–2223. doi: 10.1111/jan.15554 36565239

[ref14] Ricoeur P. (2006). Il Giudizio Medico: Morcellian.

[ref15] Santos L. B. P. A. D. Alvarenga W. A. Leite A. C. A. B. Neris R. R. Lima R. A. G. Montigny F.. (2024). Compassion fatigue: a comprehensive discussion on its development and repercussions among oncology nurses. Semin. Oncol. Nurs. 40:151655. doi: 10.1016/j.soncn.2024.151655 38782693

[ref16] SICP. Guida alla comunicazione in situazioni complesse Una guida pratica per i professionisti socio-sanitari. Accessed August 30, 2024. https://www.sicp.it/aggiornamento/linee-guida-bp-procedures/2023/09/guida-alla-comunicazione-in-situazioni-complesse/

[ref17] Stone P. Buckle P. Dolan R. Feliu J. Hui D. Laird B. J. A.. (2023). Prognostic evaluation in patients with advanced cancer in the last months of life: ESMO clinical practice guideline. ESMO Open 8:101195. doi: 10.1016/j.esmoop.2023.101195, PMID: 37087198 PMC10242351

[ref18] Tronto J. Moral boundaries: a political argument for an ethic of care. (1993).

[ref19] Tronto J. (2014). “Care: care ethics” in Bioethics. ed. Jennings B. 4th ed. Gale/Macmillan.

[ref20] Tronto J. C. Fisher B. (1990) in Toward a feminist theory of caring. eds. Abel E. Nelson M.. Suny press.

[ref21] Tuca A. Viladot M. Barrera C. Chicote M. Casablancas I. Cruz C.. (2021). Prevalence of ethical dilemmas in advanced cancer patients (secondary analysis of the PALCOM study). Support Care Cancer 29, 3667–3675. doi: 10.1007/s00520-020-05885-0, PMID: 33184713

[ref22] Tusino S. Viafora C. Furlan E. (2019) in Questioni Di Vita. Un’introduzione Alla Bioetica. ed. Angeli F.. Franco Angeli.

[ref23] Tymieniecka A. Agazzi E. (2001). “Life interpretation and the sense of illness within the human condition” in Analecta Husserliana. ed. Springer D.. Springer Dordrecht. vol. 72.

[ref24] Ullrich A. Theochari M. Bergelt C. Marx G. Woellert K. Bokemeyer C.. (2020). Ethical challenges in family caregivers of patients with advanced cancer – a qualitative study. BMC Palliat. Care 19:70. doi: 10.1186/s12904-020-00573-6, PMID: 32423444 PMC7236546

[ref25] van Ravensteijn S. G. Meijerink M. Nijenhuis-van Schayk R. Desar I. M. E. Bol K. F. van Herpen C. M. L.. (2023). The safety risk of information overload and bureaucracy in oncology clinical trial conduct. Eur. J. Cancer 183, 90–94. doi: 10.1016/j.ejca.2023.01.018 36812844

[ref26] Viafora C. (2023). La Cura e Il Rispetto—Il Senso Della Bioetica Clinica: Franco Angeli.

[ref27] Virginia H. (2006). The ethics of care: Personal, political, and global: Oxford University Press Inc.

