# Supplementary figures and images for: Ethical issues in communication in a tertiary oncology center: exploratory survey
Source: Front Psychol. 2025 Jun 6;16:1576369. doi: 10.3389/fpsyg.2025.1576369 (PMC12180531; doi:10.3389/fpsyg.2025.1576369)

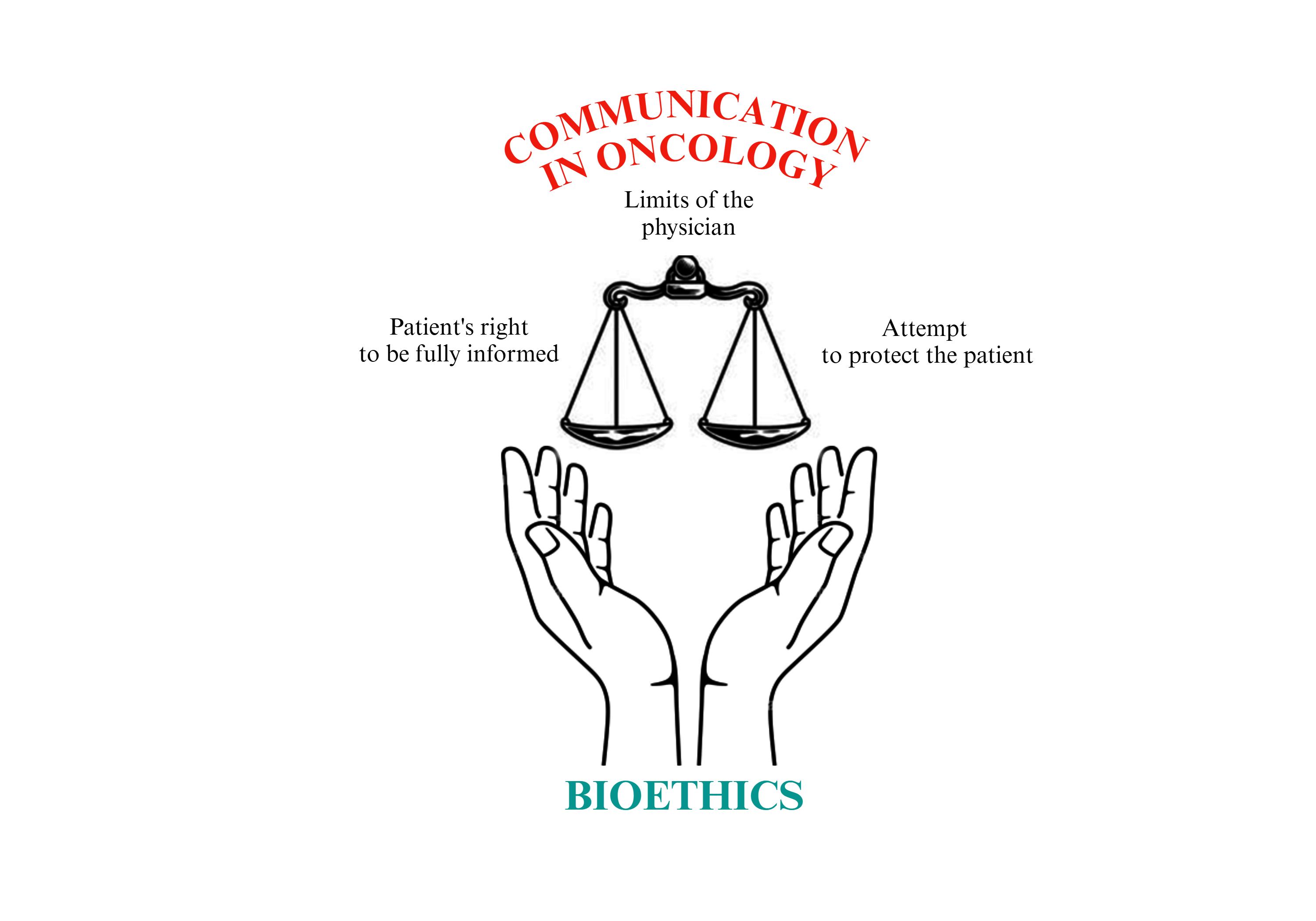

Supplement: Supplementary file 1 [file Image_1.PNG]
